# New Appliances and Things Medical

**Published:** 1902-06-07

**Authors:** 


### NEW APPLIANCES AND THINGS MEDICAL.

[We shall be glad to receive at our Office, 28 & 29 Southampton Street, Strand, London, W.C., from the manufacturers, specimens of all new preparations and appliances which may be brought out from time to time.]

### MALTOVA.

(MALTOVA FOOD COMPANY, 11 NEW STATION STREET LEEDS.)

THIS new food preparation contains the nutritive and digestive properties of a malt extract combined with the various elements which are contained in the yolk and the white of eggs. The method of preparation of Maltova is such that, while a high degree of concentration is ensured, the nutritive properties of the ingredients are in no way interfered with; the concentration is carried out "in vacuo," and the resulting mixture contains the natural enzymes of malt in a state of great efficiency. Maltova must therefore be regarded as an adjutant to digestion and a food of great nutritive value. The lecithin which is contained in yolk, of egg is now recognised as an organic body of very valuable properties in the treatment of nervous and constitutional diseases, and, in conjunction with the proteid elements of egg and the carbohydrate elements of extract of malt, it constitutes a preparation which should be valuable in the building up of new tissues and the repair of those which are wasted from fevers and other debilitating causes. Maltova is very pleasant to the taste, and will be much appreciated by children, for whose consumption it is particularly indicated.

### VIENNA FOOD.

(VIENNA FOOD CO., 9 ROSOMAN STREET, LONDON, E.C.)

This food is a highly nutritious preparation, containing carbohydrate and nitrogenous elements which are well suited to the requirements of infants and young children. It also contains salts necessary for the physiological processes of growing tissues. It is peculiarly pleasant in taste and free from all impurities; it is also sufficiently stimulating to the bowels to correct that tendency to constipation which is so usual with hand-reared infants. If Vienna Food be employed as a supplementary addition to diluted cow's milk, and in quantities suited to the individual needs of the infant, it will be found a most reliable and satisfactory preparation.

